# Can physical activity reduce excessive gestational weight gain? Findings from a Chinese urban pregnant women cohort study

**DOI:** 10.1186/1479-5868-9-12

**Published:** 2012-02-09

**Authors:** Hong Jiang, Xu Qian, Mu Li, Henry Lynn, Yanyan Fan, Hongyi Jiang, Fengling He, Gengsheng He

**Affiliations:** 1 Department of Maternal and Child Health, School of Public Health, Key Laboratory of Public Health Safety, Ministry of Education, Fudan University, Shanghai, China; 2 Sydney School of Public Health, the University of Sydney, Sydney, Australia; 3 Department of Biostatistics and Social Medicine, School of Public Health, Key Laboratory of Public Health Safety, Ministry of Education, Fudan University, Shanghai, China; 4 Department of Scientific Research and Teaching, Maternal and Child Health Care Hospital, Changzhou Municipality, China; 5 Department of Woman Nutrition Care, Maternal and Child Health Care Hospital, Changzhou Municipality, China; 6 Department of Obstetrics, Maternal and Child Health Care Hospital, Changzhou Municipality, China; 7 Department of Nutrition and Food Hygiene, School of Public Health, Key Laboratory of Public Health Safety, Ministry of Education, Fudan University, Shanghai, China

**Keywords:** Physical activity, Step count, Excessive, Gestational weight gain, Pregnant women

## Abstract

**Background:**

Excessive gestational weight gain (GWG) poses negative impact on mothers and their children. It is important to understand the modifiable lifestyle factors associated with excessive GWG during pregnancy to guide future public health practice.

**Aim:**

To investigate the association between physical activity during pregnancy and GWG of Chinese urban pregnant women.

**Methods:**

A pregnant women cohort was established between 2005 and 2007 in Changzhou, China. Physical activity levels of pregnant women were assessed using pedometer in the 2^nd ^and 3^rd ^trimester, respectively. According to step counts, pregnant women were categorized into 4 different physical activity groups: Sedentary, Low Active, Somewhat Active and Active. The pregnant women were followed for eligibility and data collection from the 2^nd ^trimester to delivery. Multiple linear regression and multiple binary logistic model were applied to determine the association between physical activity and GWG.

**Results:**

Physical activity levels and GWG of 862 pregnant women were assessed, among them 473 (54.9%) experienced excessive GWG. The adjusted odds ratio (OR) was 0.59 (95%CI: 0.36 ~ 0.95) for excessive GWG in the Active group during the 2^nd ^trimester and 0.66 (95%CI: 0.43 ~ 1.00) in the Somewhat Active group during the 3^rd ^trimester, compared with the Sedentary group respectively. In the last two trimesters, the Active group had 1.45 kg less GWG, than the Sedentary group. The ORs of excessive GWG decreased with the increased level of physical activity (*P *< 0.05).

**Conclusion:**

This study suggests that pregnant women being physically active have less weight gain during pregnancy.

## Background

Excessive gestational weight gain (GWG) is a public health concern in both developed and developing countries. Women who gain excessive weight during pregnancy are more likely to develop gestational diabetes mellitus [1], deliver a baby by caesarean section (CS) [2], become obese postpartum, and put their child at a higher risk of childhood overweight or obesity [3].

China is the most populous country in the world with more than ten million live births every year [4]. The rapid economic development in recent years has led to rapidly increased applications of automated system and household appliances. As a result, the level of physical activity of women during work or at home has decreased considerably [5]. The positive effects of gestational physical activity on the health of women and their offspring have been well recognized [6-8]. However, the association between physical activity and GWG from randomised controlled trials were still inconclusive [9-12]. In addition, most of previous studies were based on self-reported physical activity, rather than objective measures [13,14]. The aim of the study was to examine the association between physical activity during pregnancy and GWG, using an objective measurement in a large Chinese pregnant women cohort.

## Methods

### Study design

This study was part of a pregnant women cohort study conducted in Changzhou, Jiangsu Province, China between 2005 and 2007. The study was approved by the institutional review board of the School of Public Health, Fudan University, China.

### Participants and recruitment

From November 2005 through October 2007, 919 pregnant women were recruited during the 2^nd ^trimester at an antenatal clinic of Changzhou Maternal and Child Health Care Hospital, a tertiary medical institution. Pregnant women were eligible to participate if they were over 20 years old in a singleton pregnancy, and had no disease including gestational diabetes mellitus, hypertension, heart disease, chronic renal disease, and other diseases restricting physical activity. All participants had read and signed an informed consent.

### Data collection and follow-up

At the recruitment participants were asked to complete a structured questionnaire about the demographics and health related information. For assessing the physical activity levels, they were required to wear a pedometer (Omron HJ-005) for 4 days in the 2^nd ^trimester (range 18-28 weeks) and repeat the procedure in the 3^rd ^trimester (range 29-35 weeks). The recommended duration for physical activity recording included 2 working days and 2 weekend days. The Omron pedometer has been shown to have a reasonable accuracy in measuring step counts [15]. Participants were instructed to attach the pedometer on the waist of pants about 10 cm on the right or left side from the front centre of the waist. The pedometer was set to zero from the moment they got up in the morning; then to detach it and record readings before they went to bed each night. Researchers followed up the participants by telephone to answer questions encountered about using the instrument and provide required support during the study.

Data on food energy intake was collected by a 24 hour food recall at two time points, at the recruitment in the 2^nd ^trimester and in the 3^rd ^trimester, which coincided with pedometer physical activity recording.

Pre-pregnancy weight was obtained from the antenatal examination card record, which was self-reported by women when they registered their pregnancy at 1^st ^trimester. The pre-birth weight was collected from the medical records, which was obtained by measuring the actual weight at the hospital delivery unit.

### Sample size estimation

The Body Mass Index (BMI) was calculated as weight (kg)/height^2 ^(metres^2^). Overweight or obese was defined as pre-pregnancy BMI ≥ 25 kg/m^2 ^[16]. As there is no specific definition of excessive GWG for Chinese pregnant women, we defined excessive GWG as weight gain greater than the upper limits of BMI-specific weight gain recommend by the 2009 Institute of Medicine/National Research Council guidelines for weight gain during pregnancy [17]. The guidelines recommend that women with a pre-pregnancy BMI < 18.5 kg/m^2 ^should gain 12.5-18.0 kg; those with a BMI of 18.5-24.9 kg/m^2 ^should gain 11.5-16.0 kg; those with a BMI of 25.0-29.9 kg/m^2 ^should gain 7.0-11.5 kg; and those with a BMI > 30 kg/m^2 ^should gain 5.0-9.0 kg [17].

Based on the criteria proposed by Tudor-Locke [18], the pregnant women were divided into 4 active levels according to the daily step counts: Sedentary (< 5000 daily steps), Low Active (5000~7500 daily steps), Somewhat Active (7500~10000 daily steps), Active (≥ 10000 daily steps) groups, in both the 2^nd ^and 3^rd ^trimester.

The sample size calculation was based on two-sample t-tests. The estimated mean GWG for the sedentary group was 18.0 kg. The estimated differences of GWG for the Low Active, Somewhat Active, or Active groups were 1.1 kg, 1.8 kg and 2.5 kg, compared with the Sedentary group, with an estimated SD of 4.0 kg [19]. Based on this estimation a total of 826 pregnant women were needed to show a difference between the 3 groups with various activity levels and the control (Sedentary) group with a power of 90% and 95% confidence level and 15% estimated dropout rate.

### Data analysis

A Nutrition Calculation Software developed by the China Centre for Disease Control and Prevention was applied to calculate energy intake of each participant. The average daily food energy intake of 2^nd ^and 3^rd ^trimester was calculated by dividing the sum of 2 day's food energy intake values by 2.

One-way ANOVA was used to determine differences in continuous outcomes among the 4 physical activity groups, while the Pearson chi-square test and Cochran-Mantel-Haenszel chi-square test were used for categorical outcomes. Unadjusted odds ratios assessing the likelihood of having excessive versus adequate and inadequate GWG were calculated for the 4 groups.

Multiple linear regression was used to estimate the association of physical activity during pregnancy and GWG after controlling for potential confounding factors that were directly or indirectly associated with GWG [20]. These factors included mother's age, educational level, job type, the families' income, pre-pregnancy BMI, gestational age, newborns sex, passive tobacco exposure and food energy intake. Furthermore, the association between physical activity during pregnancy and excessive GWG was determined by multiple binary logistic regression after controlling for the above mentioned potential confounding factors. In addition, levels of physical activity during pregnancy were considered as an ordinal variable (coded as 1 through 4) in the logistic model, and a test of linear trend was performed. Statistical Package for Social Sciences (SPSS) for windows version 15.0 was used for all data analysis.

## Results

### Maternal characteristics according to physical activity levels

Physical activity and GWG data were obtained from 862 participants. The mean age of participants was 27 years (range 20-35). Nearly 70 percent of participants had college or above educational level and were employed in office work (light physical activities at work). Nearly 40% of participants reported monthly household income of 4,000 RMB or above. Only one woman reported smoking during pregnancy, but about half of the participants exposed to passive smoking from their partners. The mean of pre-pregnancy BMI was 20.2 kg/m^2 ^(range 14.7-28.6 kg/m^2^) and 3.4% women (29/862) were classified as overweight or obese. The median of gestational duration was 39.4 weeks. The proportion of macrosomia and Caesarean Section (CS) were 11.0% and 68.8%, respectively. There was no statistically significant difference among 4 physical activity groups on demographic and health related characteristics, except family income (Table 1).

**Table 1:** The demographic and the health related characteristics among physical activity groups of the last 2 trimesters (n = 862)

			Sedentary	Low active	Somewhat active	Active	*P*
			(n = 149)	(n = 343)	(n = 252)	(n = 118)	
**Age **(year)($\bar{x}\pm SD$)			27.2 ± 2.5	27.3 ± 2.5	26.8 ± 2.1	27.1 ± 2.8	0.15^†^
**Pre-pregnancy height **(cm) ($\bar{x}\pm SD$)			162 ± 5	161 ± 4	161 ± 4	161 ± 5	0.81^†^
**Pre-pregnancy body weight **(kg) ($\bar{x}\pm SD$)			53.5 ± 7.3	52.7 ± 6.6	52.2 ± 6.4	51.3 ± 6.1	0.05^†^*
**Pre-pregnancy BMI**(kg/m^2^)($\bar{x}\pm SD$)			20.5 ± 2.5	20.3 ± 2.3	20.1 ± 2.2	19.8 ± 2.1	0.07^†^
**Food energy intake **(kcal)/d ($\bar{x}\pm SD$)			2507 ± 228	2520 ± 241	2490 ± 247	2527 ± 257	0.40^†^
**Gestational age **(week) ($\bar{x}\pm SD$)			39.6 ± 1.0	39.4 ± 1.2	39.5 ± 1.0	39.5 ± 1.3	0.36^†^
**Education **n(%)	Junior	middle school	7(18.9)	14(37.8)	8(21.6)	8(21.6)	0.92^‡^
	Senior		15(16.9)	36(40.4)	23(25.8)	15(16.9)	
	Vocational		23(18.5)	49(39.5)	34(27.4)	18(14.5)	
	College		56(17.3)	131(40.6)	101(31.3)	35(10.8)	
	University		48(16.6)	113(39.1)	86(29.8)	42(14.5)	
**Job type **n (%)	Office work		95(15.9)	241(40.4)	183(30.7)	77(12.9)	0.17^#^
	Non office work		22(21.4)	41(39.8)	20(19.4)	20(19.4)	
	Others		32(19.6)	61(37.4)	49(30.1)	21(12.9)	
**Family Income **n (%)	≤ 4000(RMB*)		80(15.2)	212(40.2)	153(29.0)	82(15.6)	0.01^‡,^*
	4000 ~ 8000 (RMB*)		56(19.6)	107(37.5)	92(32.3)	30(10.5)	
	> 8000 (RMB*)		13(26.0)	24(48.0)	7(14.0)	6(12.0)	
**Partner smokes **n (%)	Yes		71(17.4)	165(40.5)	116(28.5)	55(13.5)	0.96^#^
	No		78(17.1)	178(39.1)	136(29.9)	63(13.8)	

^†^One-way ANOVA; ^‡ ^Cochran-Mantel-Haenszel chi-square; #Pearson chi-square *P < 0.05

### Physical activity during pregnancy associated with GWG

The participants were divided into 4 physical activity groups based on step-counts (Table 2). Among the 862 pregnant women, 473 (54.9%) experienced an excessive GWG; the least weight gain was 4 kg and the most weight gain was 37 kg. The median of weight gain was 17 kg and the mean was 17.53 kg (SD = 4.62).

**Table 2:** Steps taken by pregnant women based on physical activity levels (n = 862)

Groups	Steps					
	
	The 2^nd ^trimester		The 3^rd ^trimester		The last 2 trimesters	
	$\bar{x}\pm SD$	n(%)	$\bar{x}\pm SD$	n(%)	$\bar{x}\pm SD$	n(%)
Sedentary	3869 ± 826	155(18.0)	3713 ± 942	215 (24.9)	4086 ± 780	149(17.3)
Low active	6285 ± 684	268(31.1)	6319 ± 751	347(40.3)	6221 ± 736	343(39.8)
Somewhat	8508 ± 703	282(32.7)	8555 ± 754	209(24.2)	8413 ± 736	252(29.2)
Active	11454 ± 1252	157(18.2)	11058 ± 1028	91(10.6)	10769 ± 699	118(13.7)

**Figure 1:**
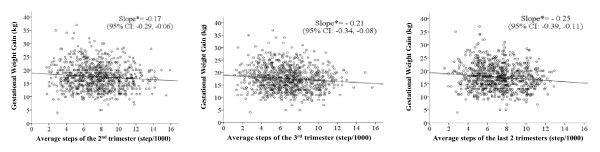
**Step counts during pregnancy in relation to gestational weight gain**. * *P *< 0.01. The slope implied that to increase every 1000 steps in the 2^nd ^trimester, 3^rd ^trimester, and the last 2 trimesters, the GWG would be reduced by 0.17 kg, 0.21 kg and 0.25 kg respectively.

The physical activity measured by step-counts was inversely related to GWG (slope = -0.17, 95%CI:-0.29 ~ -0.06, *P *< 0.01 in the 2^nd ^trimester; slope = -0.21, 95%CI:-0.34 ~ -0.08, *P *< 0.01 in the 3^rd ^trimester; slope = -0.25, 95%CI:-0.39 ~ -0.11, *P *< 0.001 in the last 2 trimesters combined, Figure 1). There were statistically significant differences of GWG among the 4 physical activity groups in the 2^nd ^trimester (*P *= 0.01), the 3^rd ^trimester (*P *= 0.02) and the last 2 trimesters (*P *= 0.02). Table 3 shows that the Active group had significantly lower maternal weight gain than the Sedentary group. No significant GWG differences were found between the Low Active group or the Somewhat Active group and the Sedentary groups. The Active group during pregnancy gained 1.45 kg less weight than the Sedentary group classified by the average physical activity in the 2^nd ^and 3^rd ^trimester.

**Table 3:** Multiple linear regression of the association of physical activity during pregnancy with gestational weight gain ^† ^(n = 862)

	Groups	Weight gain (kg)	Slope (95% CI)	*P*
		Mean ± SD		
2^nd ^trimester	Sedentary	18.05 ± 5.12		-
	Low active	18.04 ± 4.70	-0.02(-0.88,0.83)	0.96
	Somewhat active	17.26 ± 4.45	-0.85(-1.69,0.00)	0.05
	Active	16.66 ± 4.07	-1.41(-2.37, -0.45)	< 0.001
3^rd ^trimester	Sedentary	18.13 ± 4.75		-
	Low active	17.65 ± 4.70	-0.52(-1.24,0.20)	0.16
	Somewhat active	17.20 ± 4.58	-0.81(-1.62.0.01)	0.05
	Active	16.43 ± 3.79	-1.62(-2.66,-0.57)	< 0.001
Last 2 trimesters	Sedentary	17.69 ± 4.98		-
	Low active	18.04 ± 4.61	0.18(-0.60,0.96)	0.65
	Somewhat active	17.16 ± 4.52	-0.47(-1.29,0.36)	0.27
	Active	16.64 ± 4.18	-1.45(-2.44,-0.46)	< 0.001

^† ^Multiple linear regression controlling for maternal age, education, job type, family income, pre-pregnancy BMI, gestational age, newborns sex, passive tobacco exposure, daily food energy intake. Sedentary group was the reference.

### Physical activity during pregnancy associated with excessive GWG

Table 4 shows that women who were physically active had a lower risk of excessive GWG than those who were in the Sedentary group. In the 2^nd ^trimester, the Active group had a lower rate of excessive GWG, by around 40%, compared with the Sedentary group. No statistical difference was found in GWG between the Low Active or the Somewhat Active groups and the Sedentary group; while in the 3^rd ^trimester only the Somewhat Active group showed a statistically reduced rate of excessive GWG by around 35% compared with the Sedentary group. Despite of the above comparisons between groups, an opposite trend between levels of physical activity during pregnancy and the risk of excessive GWG were found. The *P *values for trend were 0.013 for the 2^nd ^trimester, 0.036 for the 3^rd ^trimester, and 0.036 for the last 2 trimesters combined. In addition, pre-pregnancy BMI was negatively associated with GWG and the daily food energy intake was positively related to the GWG.

**Table 4:** Multiple binary logistic regression of the association of physical activity during pregnancy and excessive gestational weight gain (n = 862)

	Groups	OR	**aOR**^**†**^	**95%CI**^**†**^	***P***^**†**^
2^nd ^trimester	Sedentary	1.00	1.00	-	-
	Low active	0.90	0.95	(0.62,1.46)	0.81
	Somewhat active	0.74	0.77	(0.50,1.17)	0.22
	Active	0.56	0.59	(0.36,0.95)	0.03
3^rd ^trimester	Sedentary	1.00	1.00	-	-
	Low active	0.77	0.72	(0.50,1.05)	0.09
	Somewhat active	0.65	0.66	(0.43,1.00)	0.05
	Active	0.59	0.62	(0.36,1.06)	0.08
Last 2 trimesters	Sedentary	1.00	1.00	-	-
	Low active	1.05	1.05	(0.68,1.62)	0.83
	Somewhat active	0.78	0.85	(0.54,1.34)	0.49
	Active	0.64	0.60	(0.35,1.03)	0.06

^† ^Adjusted odds ratios in multiple binary logistic regression controlling for maternal age, education, job type, family income, pre-pregnancy BMI, gestational age, newborns sex, passive tobacco exposure, daily food energy intake. Sedentary group was the reference

Furthermore, physical activity during pregnancy was positively associated with weight gain in women who had excessive GWG. Using multiple linear regression, among those women with excessive GWG, the Active group had 1.1 kg (*P *= 0.04) and 1.4 kg (*P *= 0.02) less GWG, than the Sedentary group in the 2^nd ^and 3^rd ^trimester respectively. In the last two trimesters, the Active group had 1.1 kg (*P *= 0.06) less GWG, than the Sedentary group.

## Discussion

In this cohort over 50% of pregnant women had excess weight gain during pregnancy based on the criteria derived from the new guidelines of the Institute of Medicine in 2009 [17]. Around 50% of pregnant women in the 2^nd ^trimester and above 60% pregnant women in the 3^rd ^trimester had lower levels of physical activity. The results showed that either in the 2^nd ^trimester or the 3^rd ^trimester, physical activity during pregnancy was associated with the GWG. Remaining physically active during pregnancy is associated with reduced risk of excessive GWG. To our knowledge, this is the first large cohort study using pedometer, an objective measure, to observe the association of gestational physical activity and pregnant outcome.

The average weight gains found in these participants were comparable to a Chinese study in Beijing, which showed an average GWG of 17.12 kg (SD = 4.99) [21]. The prevalence of excessive GWG of the pregnant women in this study was similar to the findings in America (51%) and Norway (55%) [13,22]. We also found that the universal excessive GWG, high prevalence of macrosomia and CS coexisted in our study, and there was a positive correlation between the excessive GWG and these undesirable pregnant outcomes (data not shown). This phenomenon calls for the urgent intervention to prevent universal excessive GWG and reduce the related negative pregnant outcomes.

The effects of physical activity on decreasing GWG are evident in both the 2^nd ^and 3^rd ^trimesters. Women in the Active group had on average 1.4 to 1.6 kg less gain in gestational weight compared with the Sedentary group, which corresponds to near 10% reduction of the average GWG of this study population. In terms of the risk of excessive GWG, women in the Active group had an average 40% less risk compared to those in the Sedentary group. Although, the results for the association in the 3^rd ^trimester or the last two trimester do not reach a 5% statistical significance level, the results indicate that active physical activity is associated with a lower risk of GWG since the majority of the range of the 95% confidence intervals is below one. The adverse effects of extra weight gain during pregnancy have been well documented [2]; the reduction of excessive GWG could potentially improve maternal and neonatal outcomes.

The association between physical activity and GWG found in our study are consistent with published works. A cohort study with more than 1,300 participants found either mid-pregnancy walking or vigorous physical activity was inversely associated with excessive GWG [22]. Another cohort study involving 622 healthy women reported that decreased physical activity during pregnancy was associated with around 2.74 lb more GWG than those maintaining or increasing physical activity during pregnancy [19]. However, Cavalcante et al. and Clapp et al. did not find significant difference in maternal weight gain between women who attend exercise and those not engaged in physical exercise during pregnancy [10,23]. One of the explanations of the discrepancy is that different definition and/or different measurement of physical activity were used in these studies. Furthermore, the small sample size of some of the studies could also result in the insufficient power to detect the difference between the intervention and control groups.

Few previous studies with large sample size had applied objective measurement to investigate the association between physical activity and pregnant outcomes. Since walking is the most common activity during pregnancy [13,24], the physical activity in this study was derived from step counts by pedometer, an objective measurement. Pedometer is easy to use, convenient to record results and inexpensive (compared with the accelerometer), thus was appropriate to monitor physical activity in a large population range including pregnant women [25]. Other strengths of our study also include that physical activity levels of pregnant women were assessed in both the 2^nd ^and 3^rd ^trimester. Four days of physical activity record by pedometer could provide a sufficient estimate of physical activity of participants in this study [18]. In addition, food energy intake had been included as a confounding factor in this study as it is an important factor of the energy metabolism.

The study has several limitations. The large proportion of well educated women in this study could limit its external validity. Some possible confounding factors, such as first-trimester nausea, glucose tolerance test results were not included, which may result in overestimate or underestimate of the association between physical activity during pregnancy and GWG. Self-reports could introduce some bias to the results that includes the pre-pregnancy weight and step counts. In addition, pedometers are good at measuring walking but not upper body movements. Finally, wearing the pedometer may lead to increased awareness, and therefore to increased activity among participants that may not reflect their true routine activities.

A recent meta-analysis of 12 randomised trials of physical activity interventions during pregnancy to prevent high GWG suggested that women in intervention groups experienced less GWG compared with the control groups [26]. As a modifiable lifestyle factor, physical activity during pregnancy could be promoted as a simple way to decrease the excessive GWG during pregnancy and prevent relevant negative pregnant outcomes. Most of the physical activities performed by pregnant women in this study were part of their routine daily lives, expecting mothers could reduce risk of excessive GWG through simple daily physical activities.

## Conclusion

This study suggests that prevalence of excessive GWG is high in urban China. Women who are physically active during pregnancy have reduced risk of excessive GWG. Physical activity during pregnancy should, therefore, be promoted as a feasible and cost effective option to lower excessive GWG.

## Abbreviations

GWG: Gestational weight gain; CS: Caesarean section; BMI: Body mass index; OR: Odds ratio; 95% CI: 95 percent confidence interval.

## Competing interests

The authors declare that they have no competing interests.

## Authors' contributions

GH, XQ, ML and HJ contributed to the study protocol and grant applications for the study; YF, HJ, FH conducted the data collection. HJ undertook analyses reported in the paper. All authors contributed to the interpretation of the data and the writing of the manuscript. All authors read and approved the final manuscript.
